# Profiling Siglec-7 and Siglec-9 ligands across the LuCaP PDX series: Implications for glyco-immune checkpoint inhibition in advanced prostate cancer

**DOI:** 10.64898/2026.09.02.748828

**Published:** 2026-10-19

**Authors:** Ziqian Peng, Libby Blencoe, Kirsty Hodgson, Maggie Orozco Moreno, Lizhi Cao, Wayne Gatlin, Li Peng, James Broderick, Richard Beatson, Jennifer Munkley

## Abstract

Immunotherapies have shown limited efficacy in prostate cancer, largely due to its immunosuppressive tumour microenvironment. Hypersialylation contributes to immune evasion by engaging sialic acid-binding immunoglobulin-like lectins (Siglecs) on immune cells, forming glyco-immune checkpoints. Although this pathway represents a promising therapeutic target, the distribution of Siglec ligands across diverse prostate cancer phenotypes remains poorly understood. Here, we utilised high-affinity engineered sialoglycan-binding reagents (HYDRA) to perform immunohistochemical profiling of Siglec-7/9 ligands across a panel of 40 LuCaP patient-derived xenograft (PDX) models. Ligand expression was evaluated in relation to AR status and neuroendocrine phenotype. To determine the impact of androgen deprivation on the tumour glycome, ligand expression was compared between matched PDX lines grown in intact and castrated mice. Our findings reveal widespread but heterogeneous expression of Siglec-7/9 ligands across the LuCaP cohort. Expression levels were comparable between AR-positive adenocarcinoma models and AR-negative neuroendocrine variants, demonstrating that this glyco-immune checkpoint is maintained across distinct prostate cancer lineages. Under castration conditions, glycan remodelling occurred in a model-dependent manner. To evaluate these therapeutic dynamics in human disease, individual trajectory analysis of paired clinical specimens from the Movember GAP1-UTMA cohort was performed, revealing patient-specific responses that phenocopy the model-dependent glycan remodelling observed across the LuCaP lines. These findings support the sialoglycan-Siglec axis as a lineage-independent target and suggest that strategies aimed at disrupting Siglec-mediated immune suppression may be most effective when combined with androgen deprivation therapy to enhance anti-tumour immunity.

